# Food-Safety Hazards in the Pork Chain in Nagaland, North East India: Implications for Human Health

**DOI:** 10.3390/ijerph110100403

**Published:** 2013-12-24

**Authors:** Anna Sophie Fahrion, Lanu Jamir, Kenivole Richa, Sonuwara Begum, Vilatuo Rutsa, Simon Ao, Varijaksha P. Padmakumar, Ram Pratim Deka, Delia Grace

**Affiliations:** 1 Veterinary Public Health Institute, Vetsuisse Faculty, University of Bern, Bern 3097, Switzerland; 2 Department of Veterinary and Animal Health, Nagaland 797001, India; E-Mails: simonao2004@yahoo.com; 3 ILRI International Livestock Research Institute, Nairobi 00100, Kenya; E-Mails: kenny.angami@gmail.com (K.R.); V.Padmakumar@cgiar.org (V.P.P.); r.deka@cgiar.org (R.P.D.); D.GRACE@cgiar.org (D.G.); 4 NRC National Research Institute on Mithun, Jharnapani, Nagaland 797106, India; E-Mail: sonuwarabegum@yahoo.com; 5 NEPED, Kohima, Nagaland 797001, India; E-Mail: vrutsa@gmail.com

**Keywords:** food-borne disease, pork, Nagaland, bacterial counts, *Taenia solium*, antibiotic residues

## Abstract

Pork occupies an important place in the diet of the population of Nagaland, one of the North East Indian states. We carried out a pilot study along the pork meat production chain, from live animal to end consumer. The goal was to obtain information about the presence of selected food borne hazards in pork in order to assess the risk deriving from these hazards to the health of the local consumers and make recommendations for improving food safety. A secondary objective was to evaluate the utility of risk-based approaches to food safety in an informal food system. We investigated samples from pigs and pork sourced at slaughter in urban and rural environments, and at retail, to assess a selection of food-borne hazards. In addition, consumer exposure was characterized using information about hygiene and practices related to handling and preparing pork. A qualitative hazard characterization, exposure assessment and hazard characterization for three representative hazards or hazard proxies, namely Enterobacteriaceae, *T. solium* cysticercosis and antibiotic residues, is presented. Several important potential food-borne pathogens are reported for the first time including *Listeria* spp. and *Brucella suis*. This descriptive pilot study is the first risk-based assessment of food safety in Nagaland. We also characterise possible interventions to be addressed by policy makers, and supply data to inform future risk assessments.

## 1. Introduction

Food and water-borne diseases are among the most important causes of sickness and death worldwide. Most human diseases are shared with animals, and food of animal origin is among the most likely to cause gastro-intestinal disease [1]. Biological hazards are usually the most important in terms of human disease burden; other hazards that may be present in meat include chemical contaminants and antibiotic residues. Even though data on the incidence of food borne disease is lacking in developing countries [2], food-borne disease is believed to be a major and widespread problem [3]. 

**Figure 1:**
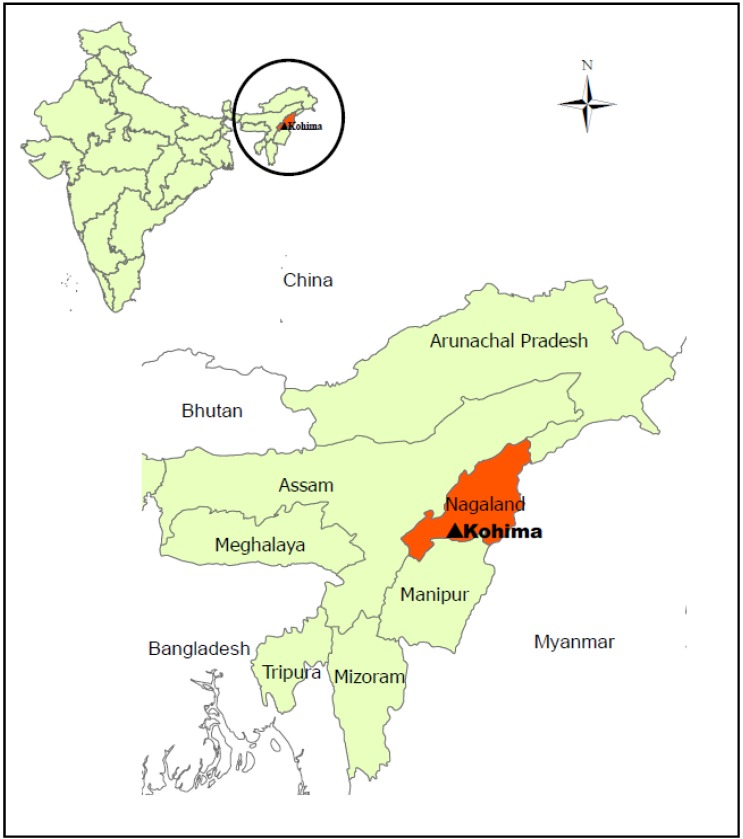
Situation map of North East India, Nagaland and Kohima, Nagaland’s capital.

Very little information is available about the situation in Nagaland, one of the states in the remote North East of India, a region geographically separated from the rest of the country (Figure 1). It is one of the smallest states in India with a geographical area of 16,579 km^2^. Agriculture (mainly practiced under the slash and burn method in hilly terrain) and livestock rearing (mainly pigs) are the main (90%) livelihood activity for the people of Nagaland followed by handlooms and handicrafts. Unlike the great majority of the Indian population who practice Hinduism, a majority (90%) of the 2 million Naga population is Christian [4]. The geographic, religious and distinct cultural traditions have influenced the food consumption habits of the population; in particular pork plays an important role in the Naga diet and is the meat most consumed [5]. Deka and Thorpe [6] found that pig production in Nagaland is small scale, with low productivity unable to satisfy the increasing local demand for pork. Thus, the market is currently dependent on supply from outside the state. The authors suggested targeted interventions to intensify the sub-sector’s productivity and to facilitate market access for smallscale local producers. One conclusion of the study was that the safety of pork needed improvement because of unhygienic practices and lengthening of the supply chain driven by population growth, urbanization and economic development [6].

Based on these recommendations, we carried out an exploratory study to estimate contamination with selected hazards and hygienic indicators in slaughter pigs and retail pork sold in Nagaland’s capital Kohima and the surrounding rural areas. Adopting a risk analysis framework, we discuss some of the identified hazards and their proxies to characterize risks for pork borne disease to consumers. Risk analysis helps answer questions of interest to decision makers and consumers: *Is this a problem? How big a problem is it? What can best be done about this problem?* Risk analysis is not widely used in the informal markets of developing countries because of human and financial resource constraints. We adapted the methodology to make it more suitable for the context.

## 2. Experimental Section

### 2.1. Selection of Hazards for Investigation

We first carried out a literature review; this found only one previous study on pork borne disease in Nagaland [7]. We then developed a list of 46 pig zoonoses based on literature for India, Europe and Asia [8,9,10]. We next eliminated those zoonoses where pork borne transmission was not confirmed, reducing the list to 18 diseases. We added antibiotic residues as a chemical hazard that experts believed to occur in pigs in North Eastern India. We conducted a review of diagnostics available which could be done in a field laboratory and at low cost. Adding these criteria reduced the list to five hazards (*Staphylococcus aureus*; *Cysticercus cellulosae*; *Brucella suis*; *Fasciola* spp. and antibiotic residues) and an additional three hazard proxies (Total aerobic counts, Enterobacteriaceae, *Listeria* spp.). (We consider the last three ‘hazard proxies’, rather than hazards, because the diagnostic test suggests, but does not confirm, that pork contains microbes with potential to cause adverse health effects). We present diagnostic test results for all selected hazards and hazard proxies and a qualitative risk characterization for three representative microbial, parasitic and chemical risks.

### 2.2. Study Design, Sampling Locations

The study was carried out in November 2009 in the District of Kohima, the capital of Nagaland with a population of 315,000 [4], representing about 1/6 of Nagaland’s population. Samples were taken at two important control points of the chain:

(a) Slaughterhouses: We visited the four largest slaughter places in different areas of Kohima, and also five village slaughters performed directly on-farm. We took faecal and blood samples and lingual palpation was performed on the fresh slaughtered pigs. As well, meat samples were taken from direct sale from the fresh slaughtered pigs on farms.
(b) Butchers: We first conducted a census of all pork selling stalls in Kohima. From this list, 25 stalls were chosen by simple random sampling. Each of these stalls was visited three times. At the first visit, the aim of the study was explained to the retailers and their approval requested. The three visits of each stall were coordinated so that they took place once early (7–9 am, E), once mid-morning (11 am–1 pm, H) and once after noon (after 1 pm–2:30 pm, L) and at each time, a meat sample (50 g) was purchased from the meat pieces displayed for sale. The samples were taken by two trained local project assistants from pieces of muscle meat displayed for sale. The pieces which were for sale varied in amount and anatomic origin between sampling times and between retailers; to minimize this bias, meat was requested from the largest piece of muscle meat present.

### 2.3. Sampling and Diagnostic Testing

The diagnostic testing systems used are listed in Table 1.

(a) Blood samples were taken from the vena cava during slaughtering process in a 5 mL or 10 mL sterile syringe out of the abdominal cavity and stored in 7 mL sterile plastic tubes without additives. It was stored on ice and allowed to clot for approximately 12 h at 4 °C followed by centrifugation at ~1200 rpm for 10 min. The serum was pipetted into sterile 2 mL tubes and frozen at −18 °C until utilization in the *Brucella suis* serologic flow assay as described in the manufacturer’s instructions.
(b) Faecal samples were taken during slaughter out of the rectal part of the intestine when it had been taken out of the abdominal cavity and before it was processed further. Approx. 10 g of faeces were put in a plastic bag which was closed and stored in a cool box and later in the refrigerator. Within 48 h following the sampling, the faeces underwent a sedimentation-flotation technique and were observed under the microscope for eggs/oocysts of protozoa and helminth species (Only results for *Fasciola* spp. eggs are reported here).
(c) Lingual palpation for cysticercosis was carried out by project veterinarians in situ in fresh slaughtered animals before *rigor mortis* set in.

**Table 1:** Outcomes of diagnostic testing of slaughter pigs and retail pork carried from Kohima, Nagaland: Hazards and classification based on reference values.

Hazard /indicator	Diagnostic testing method	*n* samples positive/ *n* samples tested (%)	Quality categories ^1^	% samples per categories
***Total aerobic bacteria***	Himedia HiTouch^TM^ Flexi Plate Agar plate culture	89/91 (97.8%)	*Satisfactory: <10^4^ cfu/g*	24.7%
			*Acceptable: 10^4^–10^5^ cfu/g*	52.8%
			*Unsatisfactory: >10^5^ cfu/g*	20.2%
			*Not classifiable*	2.25%
***Enterobacteriaceae***	Himedia HiTouch^TM^ Flexi Plate Selective agar plate culture	88/91 (94.5%)	*Satisfactory: <10^2^ cfu/g*	5.7%
			*Acceptable: 10^2^–10^3^ cfu/g*	5.7%
			*Unsatisfactory: >10^3^ cfu/g*	88.6%
***Listeria spp.***	Petrifilm^®^ selective culture system	36/91 (39.6%)	*Acceptable: <10^2^ cfu/g*	97.7%
			*Unacceptable/potentially hazardous: >10^2^ cfu/g*	2.3%
***Staphylococcus aureus***	Petrifilm^®^ selective culture system	9/19 (47.4%)	*Acceptable: <10^2^ cfu/g*	10.5%
			*Unsatisfactory: <10^2^ cfu/g*	36.8%
***Antibiotic residues***	Premi^®^ Test (DSM) ^2^	4/88 (4.5%)	*Any positive unacceptable*	4.5%
***Cysticercus cellulosae***	Lingual palpation post mortem; occasional findings in meat samples	7/80 (8.8%) (palpation])	*Any positive unacceptable*	8.8%
		2/91 (2.2%) (found when cutting meat)		2.2%
***Brucella suis***	Brucella IgG flow assay (KIT Biomedical Research)	3/53 (5.6%)	*Any positive unacceptable*	5.6%
***Fasciola* spp.**	Sedimentation	18/88 (20.6%)	*No threshold set*	

Notes: ^1^ References for categories: Regulation (EC) 2073/2005 on microbiological criteria for foodstuffs: Chapter 1. Food Safety Criteria (Listeria), Chapter 2 Process hygiene criteria (aerobic counts, Enterobacteriaceae); Mataragas *et al*. (2010) Int. J Food Microbiol (Listeria); Public Health Laboratory Service Guidelines for the microbiological quality at the point of sale. PHLS ACFDP Working group. Communicable diseases and public health, Sept 2000. (UK); Bell (CH) Guidelines for fresh meat (*Staphylococcus aureus*). ^2^ Premi®Test allows screening meat for the residues of β-lactams, Cephalosporines, Macrolides, Tetracyclines, Sulphonamides, Aminoglycosides, Quinolones, Amphenicoles and Polypeptides. Premi®Test is based on the inhibition of the growth of *Bacillus stearothermophilus*.

(d) The meat samples were placed in sterile plastic bags, placed on ice and transported to the laboratory within one hour of time where they were processed immediately to give an account of bacterial colonization at sales point. Diagnostic testing using routine tests such as (selective) colony plate count methods followed the respective manufacturer’s instructions. Classification thresholds were based on internationally recognized standards (food safety criteria/process hygiene criteria) for destructive sampling techniques for pig carcasses [11] or dissected, portioned retail meat [12] (see Table 1). Meat juice needed for the microbial screening test for antibiotic residues was gained through freezing and subsequent thawing of a small piece of each meat sample.

### 2.4. Questionnaire and Direct Observation Check List

Questionnaires were administered to the 25 pork butchers. For each butcher, six consumers were systematically selected by first estimating the total number of customers in a time period (t), and then dividing this number by six and interviewing every t/6th customer. Likewise, customers of direct slaughters were interviewed for the questionnaire survey. This and additional value chain surveys using mixed methods and participatory appraisals are described in more detail in another publication (manuscript under preparation).

### 2.5. Statistical Analysis

Descriptive and statistical analyses were carried out with NCSS [13]. Four different pork sample categories (pork sampled directly following slaughter (D) and retail pork samples taken in the early morning (E), mid-morning (H), and after noon (L)) of log transformed colony forming unit (CFU) counts of total aerobic bacteria, Enterobacteriaceae, and *Listeria spp*. were compared. Differences were tested for significance with two-tailed equal variance t tests, with the significance level (α) set at 0.05. Because four different sampling time categories were tested against each other for each pathogen/hazard dataset, a Bonferroni-adjusted significance level of 0.0083 was calculated to account for the increased possibility of type-I error.

### 2.6. Risk Assessment

In the *Codex Alimentarius* risk assessment framework, risk is defined as a function of the probability of an adverse health effect and the severity of that effect, *consequential* to a hazard [14]. A hazard is defined as a biological, chemical or physical agent with the potential to cause an adverse health effect. Three hazards (or hazard proxies) are presented in a qualitative risk assessment framework (Table 2). These were chosen, based on their importance, to represent three categories of hazards: microbial, parasitic and chemical. The methodology for risk characterisation was based on the *Codex Alimentarius* adapted for informal markets in developing countries [15]. Interview results of a total of 236 consumers of pork originating from the surveyed retailers and slaughterers were consulted to add to the exposure assessment part as well as information from participatory appraisals and focus group discussions (paper under preparation).

**Table 2:** Three selected hazards found in pork in Kohima, Nagaland, displayed in a qualitative risk assessment framework.

	Hazard identification & characterization	Exposure assessment	Risk characterization
**Enterobacteriaceae**	Bacteria of this group representing hazards for human health include *Salmonella* spp., *Klebsiella* spp. and toxigenic *E. coli*. These cause a range of symptoms from diarrhoea to urinary tract and soft tissue infection and septicaemia. General morbidity to infection with clinical symptoms is moderate, mortality is low.	In the questionnaire survey, pork consumers from Nagaland indicated that boiling of meat was the common method of preparing meat (98%). Ninety-eight % of questionnaire respondents cook pork between 30 and 190 min. This means that pork is generally well cooked which will kill Enterobacteriaceae. However there are chances for cross contamination, which is most problematic for hazards where the infectious dose is very low: notably pathogenic *E. coli* strains.	Because of thorough cooking, we consider the risk of ingestion of harmful Enterobacteriaceae from cooked pork was **low** with potentially **severe** health consequences. The risk of ingesting Enterobacteriaceae on other aliments which have been cross-contaminated through pork can be estimated higher. However we did not directly investigate these processes in this study. The uncertainty of these estimates is **high**.
***Taenia solium (Cysticercus cellulosae)***	Infection with the larval stage (cysticerci) of the tapeworm *T. solium* can lead to establishment of a tapeworm in the gut. Through the ingestion of tapeworm eggs, human cysticercosis, a serious disease, develops: Inside the body, cysticerci can develop in a number of tissues; muscle tissue can be affected as well as the eyes and the central nervous system. Neurocysticercosis [16] including epilepsy [17] is the most severe form of the disease.	Proof of highly frequent presence of the parasite was found manifold within this study: through lingual palpation and detection of viable cysticerci in meat. Also, 57% of consumers reported white, rice grain like cysts in the meat they purchased. Based on this, prevalence of *T. solium* stages in pork designated for human alimentation can be estimated as high. Thorough cooking will kill cysticerci and only 1% pork consumers in the study indicated they would eat raw meat. It is common practice in Nagaland to consume smoked pork products (indicated by 43% of consumers).	The risk of ingestion of viable cysticerci from cooked meat has to be considered **low**, because meat tends to be thoroughly cooked. The direct health impacts can be estimated as **low** and the indirect health impacts as **severe** with **moderate** associated uncertainties. Regarding the possibility of infection through cysticerci in raw/smoked meat products, the uncertainties are higher.
**Antibiotic residues**	Antibiotic residues favour the development of antibiotic resistance in bacteria and can cause allergies in sensitive persons who consume meat containing residues. Prevalence of such incidents is low when searching the literature for case descriptions; but this can only be stated with a high level of uncertainty.	Of the pork samples tested in this study, 4.5% were positive and thus contained traces of antibiotic residues. Good hygiene practices and heat treatment do not eliminate the residues and thus, the consumer is not able to influence or mitigate this risk.	We considered the risk of ingestion of antibiotic residues in pork with a direct impact on consumer’s health (anaphylactic reaction) as **very low** but **severe**. We considered indirect health impacts as **moderate**. Uncertainty of these estimates was **moderate**.

## 3. Results

### 3.1. Pork Consumption in Nagaland

Butchers interviewed in Kohima estimated that households consume about 4 kg of pork per month. Offal, head and legs were sold at the same price as pork while blood was sold at around one tenth of the price of meat. There was no price difference between fat and lean meat. The major supply of pigs for Kohima butchers were the states of Utter Pradesh and Haryana, where pig numbers are high but pork consumption very low. Pork was sold at a higher price than beef or broiler chicken but at a lower price than local chicken, dog meat, sheep and goat meat, or meat from mithun (*Bos frontalis*) cattle. However, butchers reported that demand was highest for pork followed by chicken and beef, with goat and sheep meat much lower.

### 3.2. Numbers of Samples Taken and Results of Laboratory Testing

In Nagaland, all meat is sold through the informal sector: that is small kiosks and market stalls which lack refrigeration and sanitation and which are not subject to effective health inspection. We analyzed samples from four Kohima slaughter places and five village slaughters. Eighty-eight fecal samples and 88 blood samples were taken from slaughter pigs and 80 lingual palpations carried out with the fresh slaughtered pigs. Ninety-one meat samples were taken and analysed from 25 Kohima butcher retail stores (*n* = 3 each), one slaughter place with direct sale (*n* = 10) and five village slaughtered pigs. The results and quality categories compared to international standards are shown in Table 1.

Overall, 88.6% of the samples had unsatisfactory high levels of Enterobacteriaceae. In total aerobic bacteria counts, 20.2% of samples were classified unsatisfactory. We found *Listeria* spp. in 39.6% of all samples, but 97.7% of these had a concentration lower than the estimated minimal infectious dose (estimated at 10^2^ CFU/gram for *L. monocytogenes* [18] for highly susceptible individuals (pregnant women, newborns, elderly, and immunocompromised)). *Staphylococcus aureus* was detected in 47.6% of samples and most of these were at unacceptable levels. *Brucella suis* was detected in 5.6% of blood samples and *Fasciola* spp. eggs in 20.6% of fecal samples. Antibiotic residues were detected in 4.5%, and viable *T. solium* cysticerci in 2.2% of the meat samples. Further, in 8.8% of the lingual palpations, cysticerci were palpable.

**Figure 2:**
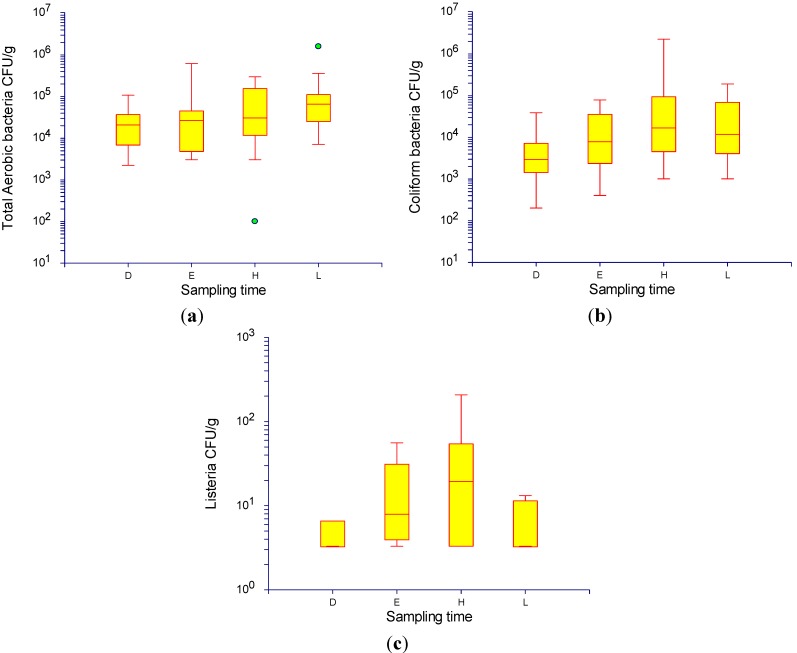
Bacterial plate counts from pork sampled directly following slaughter (D) and retail pork samples taken in the early morning (E), mid-morning (H), and after noon (L) carried out to estimate the burden of contamination in Kohima, Nagaland. (**a**) Total aerobic bacteria; (**b**) Enterobacteriaceae (coliform bacteria); (**c**) *Listeria* spp.

The quantitative differences of total aerobic bacteria, Enterobacteriaceae, and *Listeria* spp. between retail pork samples (pork sampled directly following slaughter (D) and retail pork samples taken in the early morning (E), mid-morning (H), and after noon (L)) are displayed in Figure 2. The mean log of CFU per gram pork was lower in D samples for all three tested groups of bacteria. The difference was significant in a two-tailed *t*-test (*p* < 0.0083) between D/L and E/L samples for total aerobic bacteria, and between D/H as well as D/L samples for Enterobacteriaceae.

### 3.3. Risk Assessment (See Table 2)

The first step of risk assessment is *hazard identification* (that is, identification of biological, chemical, and physical agents capable of causing adverse health effects). As summarised in Table 2, we considered Enterobacteriaceae to be indicative of important hazards because this family includes *Salmonella* spp., toxigenic *E. coli* and other bacteria, which are considered known and important hazards. The two hazards chosen (*T. solium* and antibiotic residues) have known adverse effects on human health. 

The next step of risk assessment is *hazard characterization*, which evaluates the nature of the adverse human health impacts associated with a hazard, including dose-response relations where appropriate.

1. The most common health effects of Enterobacteriaceae are gastro-intestinal illness. This is often most serious in young, old, pregnant and immune-compromised people. A minority of cases can have severe and even fatal illness (haemolytic uremia syndrome from STEC or septicaemia from salmonellosis). Only a few cells of some *Salmonella* and STEC strains are sufficient to initiate disease in susceptible humans and levels of Enterobacteriaceae were very high.
2. Ingestion of viable *T. solium* cysts can result in establishment of a tapeworm in the gut. Tapeworms can cause gastrointestinal symptoms but these are usually not severe. A more significant health impact is neurocysticercosis as the result of auto-infection. An indirect impact is when the eggs of the tapeworm are ingested by a pig which then develops cysts, allowing the cycle of the parasite to continue.
3. Antibiotic residues can directly cause allergies in sensitive persons, however, cases are quite rare. There is a potential for antibiotic residues in food to promote antibiotic resistance in bacteria pathogenic to humans. However, this has not been quantified.

The third step of risk assessment is *exposure assessment* which estimates how likely populations or individuals are to ingest hazards and at what levels.

1. We found that levels of contamination with Enterobacteriaceae were unacceptably high at all steps along the value chain. The likelihood of consumption of pathogenic organisms was reduced by the long period of cooking, but the likelihood of cross contamination in the kitchen was high as most people did not separate pork and vegetables during preparation. In addition, we found a local practice of storing pork for several weeks in the chimneys and this smoked pork was not always cooked before eating. Less than 10% of households have refrigeration and owners of refrigerators stored meat three times longer than people without. According to secondary data, mothers report 1.6 cases of diarrhoea in infants each year [19].
2. *Taenia solium* prevalence in meat and by lingual palpation were high, as has been reported for pigs in the rural North East of India [7,20]. Prolonged cooking is effective at inactivating cysts and cross-contamination is not likely to be a problem. However, the effect of traditional smoking of pork in chimneys on cyst viability is not known. Moreover, secondary data shows a high prevalence of asymptomatic and clinical neurocysticercosis in Northern India, especially in pig farming communities [21,22]. This suggests that people are exposed to tape-worm eggs which are shed by people who have consumed pork with cysts. The implication is that viable cysts are being ingested in Nagaland.
3. Antibiotic residues are not eliminated through good hygiene practices and heat treatment and thus, the consumer is not able to influence this risk. Around one in 20 pork samples had unacceptable levels which in conjunction with the high consumption of pork, suggests that exposure is common.

The final step is *risk* *characterization* which is defined as the estimation, including uncertainties, of the probability of occurrence and severity of known or potential adverse health effects in a given population based on hazard identification, hazard characterization and exposure assessment. This estimation is given in Table 2.

## 4. Discussion

### 4.1. Importance of Systematic Selection of Hazards

Within the wide range of possible hazards from pork, fewer are likely to have a major impact on public health [23]. Especially in resource poor environments, it is best to target efforts to that small group of pathogens causing the majority of disease. Therefore, one aim of the study was to develop a method to reduce the list of all hazards to a smaller number of ‘priority hazards’ which are most likely to be problematic and which could be assessed given the limited laboratory resources. This could also help to identify best-bet targets for (quantitative) risk assessments which have not often been carried out for contexts of data scarce developing counties [24]. This study is to our knowledge the first published assessment of microbiological hazards in pork in Nagaland, where pork represents an important part of the diet. 

### 4.2. Importance of Pork-Borne Disease in Nagaland

Nagaland has the highest density of pigs in India and highest pork consumption levels. We consider the estimate for the quantity of pork consumed per household to be rather high because butchers are more familiar with consumption patterns of richer households. Given the high consumption of pork our finding of generally low microbiological quality is of concern. For example, only 25% of the samples had a satisfactory low level of total aerobic bacteria indicating poor handling and poor hygiene. However, we also documented a number of practices that reduce risk including especially long cooking times and typical consumption of pork within 12 h of pig slaughter.

This study was the first to report *Listeria* spp., *B. suis* and *Fasciola* spp. eggs from swine and pork sampled in Nagaland. Infection with *Listeria monocytogenes,* an important food safety hazard and only pathogen in the genus *Listeria* spp., can lead to severe disease such as neurologic symptoms, meningitis, abortion and stillbirth. *Listeria* spp. are ubiquitous in the environment and are psychrotroph bacteria. Thus, *L. monocytogenes* is a good example for a public health hazard with a potential to increase with life style conditioned “modern” practices: Coinciding with the advent of refrigeration in retail and homes, food is stored longer, allowing *L. monocytogenes* to multiply over longer periods of time [25,26]. While cold chains, and longer storage, are not yet dominant in Nagaland, *L. monocytogenes* is likely to be among the *Listeria* spp. we found, has a potential to emerge as a risk, and should thus be kept in mind for future assessments.

*Fasciola hepatica* and *F. gigantica* belong to the plant borne trematode zoonoses. Both are present in Asia and can infect pigs and humans. Infection of pigs is very likely to occur through ingestion of parasite larvae on fresh vegetable and greens. It is likely that where animal cases are reported, human cases also exist [27]. These trematodiases result in severe liver disease and cause disability and death. The eggs of the two liver fluke species *Fasciola hepatica* and *F. gigantica* cannot be morphologically distinguished [28].

Porcine brucellosis, caused by the bacterium *Brucella suis*, is an economically important cause of reproductive losses in pigs [29]. *B. melitensis* being the most pathogenic to humans, *B. suis* can as well cause zoonotic infection with human disease, often as a result of occupational exposure [30]. It is thus a noteworthy result that *B. suis* is present in the Naga pig population.

### 4.3. Risk Mitigation

Using a structured risk assessment approach was useful in allowing hazards to be prioritized according to likely health impacts rather than levels of stakeholder concern. Our risk assessment suggested that Enterobacteriaceae was a priority issue both for further assessment and risk mitigation. Our observations and questionnaires identified the slaughter places as an important source of contamination with faeces because of processing on the floor, lack of adequate water and waste disposal, and poor practices by slaughterhouse workers. Similar problems are seen in many parts of India, where slaughter is often without running water or basic separation into a dirty and a clean compartment [31]. Substantial investments in infrastructure are often needed to assure food safety, but funding is rarely available. However, recent studies have shown that substantial and cost effective improvements in hygiene and meat safety can be achieved through training of butchers and simple innovations such as provision of protective clothing and disinfectants [32]. This study in an abattoir and associated meat market in Nigeria estimated that peer-to-peer training and providing basic hygienic equipment to butchers cost $8.82 and averted 175 cases of diarrhoea a year saving $780 in illness associated costs.

An important control point was the time between slaughter and sale. Meat sampled early in the morning was of better quality and microbial levels rapidly increased. Reducing the time to sale or improving storage and handling conditions in the butchers’ kiosks could reduce the growth of microbes. Von Holy and Makhoane [33] give examples of policy and practice innovations that improved safety of food sold in streets and kiosks in South Africa.

Another control point was poor food handling practices in the household which allow cross-contamination. Lack of refrigeration in the household may contribute to unsafe pork but those with refrigerators stored food for longer periods, which is problematic as electricity cuts are very common in Nagaland. Several studies in developing countries have shown a high level of consumer concern over food safety [34]. Hygiene training at household level is often effective [35], but the costs tend to be high and controlling contamination further down the supply chain may be preferred.

The traditional practice of storing and smoking pork in the chimney [36] requires further information to assess its impact on food-borne pathogens. In some cases traditional practices are effective at reducing food-borne pathogens, but in other cases they can increase risk [37].

## 5. Conclusions

This is the first study testing pigs and pork for a range of infectious and non-infectious hazards to consumers in Nagaland, North East India. Laboratory testing revealed substantial microbial contamination as well as antibiotic residues. Several important pathogens were identified for the first time in Nagaland. We used a risk assessment framework to assess the health impacts of three representative hazards (or hazard proxies) to generate information for decision makers. We also showed that by using participatory methods and rapid diagnostics alongside conventional methods risk assessment could be used in a resource scarce setting. However, this was a preliminary study and further research is needed to better understand, and ideally quantify, the health risk associated with hazards identified as well as to test for other potentially important hazards. We identified opportunities for risk mitigation along the value chain. We recommend training of slaughterhouse workers and butchers using peer-to-peer methods, in combination with improving consumer practices. Decision-makers need to be involved and convinced of the benefits of improving food safety in the informal pork sector. In conclusion, pork is very important to diets in Kohima, Nagaland; it is sold through the informal sector and contains high levels of hazards. However risk to human health is reduced by value chain and consumer practices.
